# Early Effects of Reward Anticipation Are Modulated by Dopaminergic Stimulation

**DOI:** 10.1371/journal.pone.0108886

**Published:** 2014-10-06

**Authors:** Thore Apitz, Nico Bunzeck

**Affiliations:** 1 Department of Systems Neuroscience, University Medical Center Hamburg-Eppendorf, Hamburg, Germany; 2 Department of Psychology, University of Lübeck, Lübeck, Germany; Centre de Neuroscience Cognitive, France

## Abstract

The abilities to predict future rewards and assess the value of reward delivery are crucial aspects of adaptive behavior. While the mesolimbic system, including dopaminergic midbrain, ventral striatum and prefrontal cortex have long been associated with reward processing, recent studies also indicate a prominent role of early visual brain regions. However, the precise underlying neural mechanisms still remain unclear. To address this issue, we presented participants with visual cues predicting rewards of high and low magnitudes and probability (2×2 factorial design), while neural activity was scanned using magnetoencephalography. Importantly, one group of participants received 150 mg of the dopamine precursor levodopa prior to the experiment, while another group received a placebo. For the placebo group, neural signals of reward probability (but not magnitude) emerged at ∼100 ms after cue presentation at occipital sensors in the event-related magnetic fields. Importantly, these probability signals were absent in the levodopa group indicating a close link. Moreover, levodopa administration reduced oscillatory power in the high (20–30 Hz) and low (13–20 Hz) beta band during both reward anticipation and delivery. Taken together, our findings indicate that visual brain regions are involved in coding prospective reward probability but not magnitude and that these effects are modulated by dopamine.

## Introduction

The ability to rapidly respond to rewarding events and to predict their occurrence is thought to be of evolutionary importance [1]. Indeed, seminal work in non-human primates has shown that dopaminergic midbrain neurons [2], [3], as well as neurons in the prefrontal cortex [4], basal ganglia [5] and parietal cortex [6] respond to cues that predict a reward as early as ∼100 ms after stimulus onset. Importantly, recent evidence indicates that the ability to predict forthcoming rewards is not only limited to the mesolimbic system (i.e. including the above mentioned structures) but also extends to the primary visual cortex (V1) [7]. Moreover, activity in visual area V4 has been found to be controlled by dopamine dependent frontal eye field activity [8], suggesting a role of dopamine in mediating neural activity in the visual cortex in response to behaviorally relevant stimuli.

In humans, functional magnetic resonance imaging (fMRI) studies have also demonstrated a prominent role of the mesolimbic system in reward processing [9]–[17]. However, only little is known about the precise temporal dynamics underlying reward processing in early visual brain regions. Using magnetoencephalography (MEG), Bunzeck et al. [18] found that reward probability is signaled at occipital sensors at a comparable speed as in non-human primates, namely at ∼100 ms. More precisely, in a monetary reward anticipation task, three cues predicted the delivery of monetary gains with different probabilities, which were coded in the event-related magnetic fields (ERFs). In line with these observations, subsequent studies demonstrated similar neural effects at ∼150 ms after stimulus onset in different reward tasks [19]. Apart from probability, there is also evidence indicating that magnitude is signaled at comparable latencies as shown in EEG (high vs. low reward predicting cues) [20] and combined EEG/MEG studies (no reward vs. reward predicting cues) [21].

In the frequency domain, reward processing has been shown to be signaled by changes in oscillatory power in the beta frequency range. Specifically, frontal beta power (20–30 Hz) has been found to increase as a function of reward probability during reward anticipation [18] and oscillatory activity in this frequency range is increased following gains compared to losses in gambling tasks [22], [23]. Similarly, beta power increased during the anticipation of a high reward vs. no reward as shown in a visual working memory task [24] suggesting that beta oscillations signal both the anticipation and outcome of monetary incentives.

These findings demonstrate that reward anticipation is underpinned by very rapid cortical neural mechanisms, and they suggest that probability and magnitude of an expected reward may already be represented at early stages of perceptual processing. Furthermore, given the engagement of dopamine [8] in indirectly modulating neural activity in visual brain regions, it appears likely that dopamine might mediate these early reward signals.

To test this hypothesis, we used MEG in healthy humans together with psychopharmacology. More specifically, participants received either the dopamine precursor levodopa or a placebo compound and subsequently performed a cued reward task in which a set of visual stimuli predicted rewards of varying magnitude and probability (2×2 factorial design). Based on previous work in humans [18], we hypothesized early effects of reward probability and magnitude over occipital regions shortly after cue presentation (i.e., ∼100–200 ms). We also predicted that these effects would be modulated by levodopa administration, which would be indicative of a role of dopamine in mediating these effects. In order to fully assess the potential impact of levodopa on reward processing, we assessed both reward anticipation and delivery.

## Methods

### Participants

38 human subjects participated in the experiment. All were randomly assigned to one of two experimental groups in a double-blind fashion. We used a between-subjects design instead of a within-subject design mainly for practical reasons since they are less prone to drop outs and training effects. Importantly, all data were analyzed using appropriate statistics (see below) to account for between subjects variance. 20 participants (ten males; age range = 21–34 years; mean age = 26.4 years; SD = 3.53 years) orally received the dopamine precursor levodopa (150 mg levodopa, 37.5 mg benserazid) prior to the experiment (‘levodopa group’) while the other 18 participants (ten males; age range = 18–33 years; mean age = 24.6 years; SD = 4.19 years) received a placebo (‘placebo group’). Levodopa is licensed for the treatment of Parkinson's disease and provokes only little to no side-effects if taken in low dosages. It has been used in previous imaging studies [25]–[28]. To reduce possible between-subject variance in the duration of the drug to take effect, all subjects were asked not to eat for the duration of 2 h before the study appointment.

All subjects were healthy, right-handed and had normal or corrected-to-normal vision. None of the participants reported a history of neurological, psychiatric, or medical disorders or any current medical problems. Subjects gave written informed consent after they were given detailed explanation of the experiment. The study received approval of the local ethics committee (Medical Council Hamburg).

### Experimental design and task

The experiment described here was part of a series of experiments (unrelated regarding task design and hypotheses, [28]) at the beginning of which drugs were administered. Therefore, the main task started 1.5 h after drug intake. Since levodopa reaches peak blood plasma concentration about 45–60 min after intake and has a half-life of approximately 80 min [29], sustained drug effects on neural processes were to be expected for the duration of the experiment. In order to assess possible side-effects, participants filled in subjective rating scales on three time points (T1: before drug administration; T2: 45 min after drug administration, and T3: at the end of the MEG experiment, ∼2 h after drug administration). No differences in subjective ratings between treatment groups were detected (for details see Tables S1, S2 and Analysis S1).

**Figure 1:**
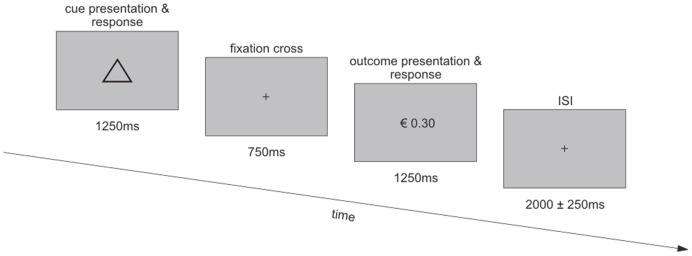
Experimental design. Visual cues predicted outcomes of high (1.00€) or low (0.30€) magnitude and high (0.7) or low (0.3) probability. Participants were initially familiarized with cue-reward associations in a conditioning phase (not shown; see methods section for details).

The task was divided into two phases. All participants performed (1) a conditioning phase followed by (2) a test phase inside the MEG scanner (Figure 1).

1. Conditioning phase: At the beginning of the experiment, four different geometrical shapes (circle, triangle, rhombus and square; in the following referred to as cues) were paired, under different probabilities (0.3, 0.7), with a monetary reward magnitude of either €0.30 or €1.00, resulting in four unique experimental conditions (probability: 0.3, reward magnitude: €0.30; probability: 0.3, reward magnitude: €1.00; probability: 0.7, reward magnitude: €0.30; probability: 0.7, reward magnitude: €1.00; i.e. 2×2 factorial design). Each cue was presented 20 times and was mapped to one of four different buttons. On each trial, one of the four cues was presented on the screen for 1250 ms, followed by the presentation of a white fixation cross for 750 ms. Participants indicated the identity of the stimulus by pressing the corresponding button using their index finger and middle finger of both their left and right hand. Responses could be made while the cue was displayed on the screen and during the following fixation period. Subsequently, the probabilistic outcome (€0.00, €0.30 or €1.00) was presented as a number on the screen for another 1250 ms and participants had to indicate whether they had won any money and if they had, how much, using their thumb, index finger and middle finger of their right hand. Here, responses could be made while the outcome was displayed on the screen and during the inter-trial interval which lasted 2000±250 ms. The conditioning phase served as a training for the participants to learn the contingencies of cues and their respective outcome probability and reward magnitude.
2. Test phase: The test phase was split into three blocks. Each block consisted of 20 presentations of each of the four cues and their probabilistic outcomes (i.e., 80 trials). Timing was identical to the conditioning phase regarding presentation times of cues, fixation cross and outcomes, as well as the duration of the inter-trial intervals. During each block, the presentation order of the four cue types was fully randomized. Participants could take self-paced pauses between blocks.

Prior to the experiment, participants were instructed to react as quickly and as correctly as possible to both cue and outcome presentation and that they would be paid their earnings (i.e. correct trials) up to €20.

Cue stimuli were black line drawings, while outcomes were presented in white numbers and letters. All stimuli were presented on a gray background (gray-value of 127, 8-bit gray-scale ranging from 0–255).

### Behavioral data analysis

Reaction times (RTs) and hit rates (proportion of correct responses to the maximum number of stimuli of one condition) during the test phase were separately analyzed for responses to cue stimuli (‘probability: 0.3, reward magnitude: €0.30’, ‘probability: 0.3, reward magnitude: €1.00’, ‘probability: 0.7, reward magnitude: €0.30’ and ‘probability: 0.7, reward magnitude: €1.00’ [four conditions]) and outcomes (‘€0.00’, ‘€0.30’ and ‘€1.00’ [three conditions]). RT scores and hit rates of these conditions were entered into a 2×2×2 (cues) and 3×2 (outcomes) analyses of variance (ANOVAs). The ANOVAs for cues comprised the within-subject factors probability (0.3, 0.7), reward magnitude (€0.30, €1.00) and the between-subject factor drug group (levodopa, placebo) and the ANOVAs for outcomes comprised within-subject factor outcome magnitude (€0.00, €0.30, €1.00) and the between-subject factor drug group (levodopa, placebo).

### MEG methods

MEG recordings took place in a magnetically shielded room via a 275-channel CTF MEG-system with SQUID-based axial gradiometers (VSM MedTech Ltd., Couquitlam, BC, Canada) and 2^nd^ order gradients. Neuromagnetic signals were continuously digitized at a sampling rate of 1200 Hz and behavioral responses were made via a MEG-compatible response pad. Data were low-pass filtered at 240 Hz during acquisition and subsequently analyzed with SPM8 (Wellcome Trust Centre for Neuroimaging, University College London, UK) and MATLAB software (The MathWorks, Inc., Natwick, MA, USA).

### ERF analysis

For the analysis of the ERFs, MEG data were high-pass filtered at 0.25 Hz and low-pass filtered at 15 Hz using Butterworth filters. Subsequently, they were extracted from 100 ms before to 1000 ms after stimulus onset and baseline corrected relative to the 100 ms before stimulus onset (epoching). Epoched data were then down-sampled at 150 Hz and artifact detection was performed using simple thresholding to remove artifact-containing trials with signals exceeding 2500 fT before averaging trials for each condition separately. Only trials with correct behavioral responses to both cue and outcome were used for averaging.

**Figure 2:**
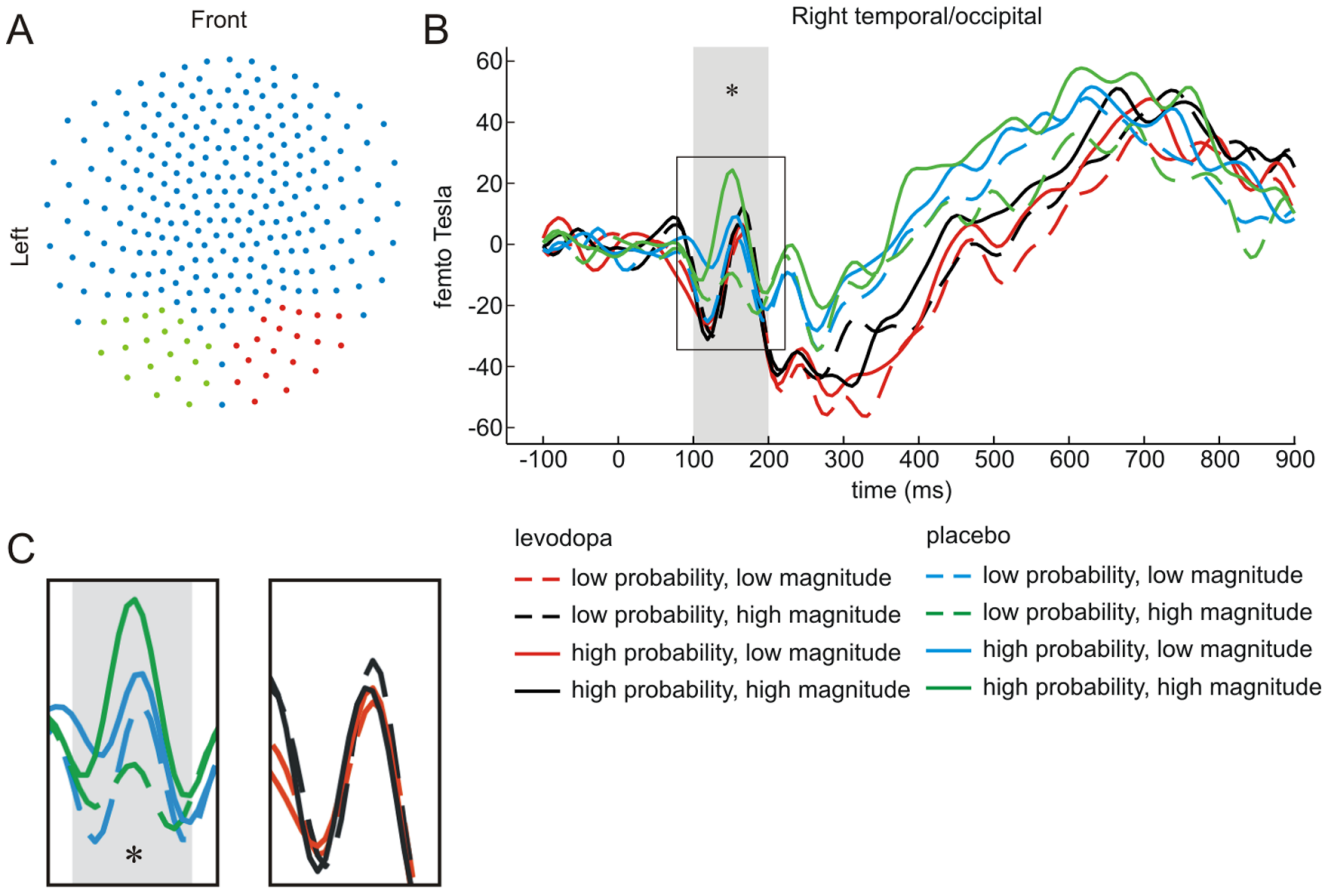
ERFs for reward predicting cues. An interaction effect between reward probability (high/0.7, low/0.3) and drug group (levodopa, placebo) was observed at a priori defined sensor groups of interest (red and green dots in A, see text) in the time window of 100 to 200 ms (B). This interaction was driven by a significant probability effect in the placebo but not levodopa group. C shows a magnification of this effect. Asterisk indicates the statistical significant interaction (*p*<0.05).

In a first step, analyses of the ERFs in response to cues were limited to two *a priori* defined clusters of bilateral occipito-temporal sensors, which were previously identified to show an effect of reward probability following a reward predicting visual cue [18]. One cluster consisted of the left hemisphere sensors MLO52, MLO42, MLO31, MLO21, MLO22, MLO32, MLO43, MLO53, MLT57, MLO44, MLO33, MLO23, MLO12, MLT47, MLO34, MLO24, MLO13, MLO41 and MLO51, while the second cluster comprised the corresponding sensors of the right hemisphere (see Figure 2A). On the basis of previous research, we focused on the time window of 100 to 200 ms after stimulus onset [18]. Averaged ERFs for each condition, participant and time window were entered into a 2×2×2×2 ANOVA with the within-subject factors hemisphere (left hemisphere, right hemisphere), probability (0.3, 0.7), reward magnitude (€0.30, €1.00) and the between-subject factor drug group (levodopa, placebo).

In a second step, less hypothesis-driven analysis, we were interested in studying the effects of levodopa on outcome responses across all sensors and time-points (i.e., the whole time window ranging from −100 ms to 1000 ms after stimulus onset). To this end, epoched and averaged data were converted into Neuroimaging Informatics Technology Initiative (NIfTI) format, producing one 3D image of channel space x time for each condition and participant. The 2D channel space was created by projecting sensor locations onto a plane followed by a linear interpolation to a 64×64 pixel grid (pixel size 2.12×2.69 mm). The time dimension consisted of 166 samples per epoch with a length of 6.67 ms each. These images were smoothed using a Gaussian kernel (full-width half-maximum, FWHM) of FWHM = 5×5×15 mm. Smoothing was done to allow for accommodating the spatial and/or temporal variance between participants. It also leads to a better conformity regarding random field theory [30].

Subsequently, the 3D images for each condition and participant were entered into a 3×2 ANOVA, which comprised the within-subject factor outcome magnitude (€0.00, €0.30, €1.00) and the between-subject factor drug group (levodopa, placebo), allowing us to test for both main effects and interactions.

### Time-frequency analysis

Time-frequency (TF) data were high-pass filtered at 4 Hz; low-pass filtered at 100 Hz; epoched from 450 ms before to 1000 ms after stimulus onset; baseline corrected relative to 450 ms before stimulus onset; down-sampled at 250 Hz and thresholded at 2500 fT. Oscillatory activity in the MEG signal was quantified by continuous Morlet wavelet transformation (factor 7). This wavelet decomposition was applied to each trial, sensor and subject across the frequency range of 4–40 Hz. This was followed by averaging across all trials of the same condition and a rescaling of the TF spectrogram by dividing the power of the trial (p) by the power in the baseline (p_b) and taking the logarithm of this ratio [LogR: (log(p/p_b))]. Rescaling of TF data was done for better visualization and should have no impact on the subsequent statistical comparison. Subsequently, the rescaled TF data were converted into NIfTI format for each of the two frequency ranges of interest (low beta: 13–20 Hz; high beta: 20–30 Hz) separately, creating 3D images of channel space x time (averaged across 13–20 Hz and 20–30 Hz, separately) [31]. Similar to the ERF analysis, the 2D channel space was created by projecting the sensor locations onto a plane followed by a linear interpolation to a 64×64 pixel grid (pixel size 2.12×2.69 mm). The time dimension consisted of 363 samples per epoch with a length of 4 ms. These images were smoothed via a Gaussian kernel of FWHD = 5×5×15.

TF data for cues and outcomes were analyzed separately via 2×2×2 (cues) or 3×2 (outcomes) ANOVAs. The ANOVAs for cues comprised the within-subject factors probability (0.3, 0.7), reward magnitude (€0.30, €1.00) and the between-subject factor drug group (levodopa, placebo) and the ANOVAs for outcomes comprised within-subject factor outcome magnitude (€0.00, €0.30, €1.00) and the between-subject factor drug group (levodopa, placebo). Statistical analyses of the TF data were limited to the time window ranging from −250 ms before to 900 ms after stimulus onset to avoid edge effects induced by Morlet wavelet transformation. Using ANOVAs to analyze TF data is well-established and a common approach [18], [32], [33].

All 2^nd^ level analyses in SPM8 were thresholded at an uncorrected level of *p*<0.001 (unless stated otherwise) followed by family-wise error (FWE) correction for multiple comparisons. Further detailed information on the methods of SPM8 for EEG and MEG data analysis can be found elsewhere [34]. MEG and behavioral data were assessed regarding distribution and variance; when the sphericity assumption was violated, Greenhouse-Geisser correction was applied accordingly.

## Results

All analyses (behavior and MEG) are based on trials with correct behavioral responses to both cue and outcome and RTs<1500 ms to cue and outcome presentation. Explorative data analysis identified three participants that exhibited outlying task performance (i.e., accuracy scores more than two standard deviations lower than the group mean) which were excluded from all analyses.

### Behavioral results – accuracy, reaction times

Behaviorally, participants discriminated between the four cues and between outcome magnitudes with high accuracy (Table 1). A 2×2×2 ANOVA on hit rates for cues revealed a main effect of probability (*F*(1,33) = 4.16, *p* = 0.049) but no main effect of magnitude (*F*(1,33) = 0.58, *p* = 0.453) or drug group (*F*(1,33) = 0.07, *p* = 0.790) and no interactions (all *p*'s>0.05). The main effect of probability was driven by higher hit rates (collapsed across groups and magnitude levels) for high probability cues (i.e., 0.7) compared to low probability cues (i.e., 0.3) (*t*(34) = 2.03, *p* = 0.050). A 3×2 ANOVA on hit rates for outcomes did not show any significant main effect for drug group (*F*(1,33) = 0.42, *p* = 0.839) or outcome magnitude (*F*(2,66) = 0.40, *p* = 0.669) and no interaction of drug group and outcome magnitude (*F*(2,66) = 2.29, *p* = 0.109). See Table 2 for details on outcome.

**Table 1:** Behavioral results anticipation.

	Levodopa	Placebo
	M (SD)	M (SD)
hit rates		
low probability, low magnitude	*0.93 (0.07)*	*0.92 (0.09)*
low probability, high magnitude	*0.93 (0.07)*	*0.94 (0.05)*
high probability, low magnitude	*0.93 (0.07)*	*0.96 (0.04)*
high probability, high magnitude	*0.95 (0.05)*	*0.94 (0.08)*
RT (in ms)		
low probability, low magnitude	*665.29 (74.03)*	*644.37 (47.81)*
low probability, high magnitude	*664.43 (73.82)*	*637.95 (53.76)*
high probability, low magnitude	*643.28 (52.94)*	*626.91 (63.19)*
high probability, high magnitude	*632.32 (63.17)*	*599.10 (58.87)*

Discrimination performance and reaction times. Mean values are shown. M: mean (SD: standard deviation).

**Table 2:** Behavioral results outcome.

	Levodopa	Placebo
	M (SD)	M (SD)
hit rates		
high reward	*0.93 (0.06)*	*0.91 (0.09)*
low reward	*0.90 (0.08)*	*0.93 (0.06)*
no reward	*0.91 (0.06)*	*0.91 (0.07)*
RT (in ms)		
high reward	*544.19 (69.79)*	*509.36 (87.29)*
low reward	*562.00 (74.84)*	*527.18 (83.98)*
no reward	*549.12 (54.28)*	*542.24 (64.01)*

Discrimination performance and reaction times. Mean values are shown. M: mean (SD: standard deviation).

RT analysis (2×2×2 ANOVA) of cue responses revealed a main effect of probability (*F*(1.33) = 19.89, *p*<0.001) but no statistically significant main effect of drug group (*F*(1,33) = 1.92, *p* = 0.175) or reward magnitude (*F*(1,33) = 3.22, *p* = 0.082) and no interactions (all *p*'s>0.05). The main effect of probability was based on faster RTs to high probability cues (i.e., 0.7) compared to low probability cues (i.e., 0.3) (*t*(34) = −4.53, *p*<0.001). For outcomes, a 3×2 ANOVA did not reveal any significant main effect of drug group (*F*(2,66) = 1.36, *p* = 0.252) or outcome magnitude (*F*(2,66) = 2.29, *p* = 0.109) and no interaction between drug group and outcome magnitude (*F*(2,66) = 1.32., *p* = 0.273). See Table 2 for details on outcome.

### MEG results – ERF responses

To assess the effects of probability, magnitude and possible interactions with drug group at occipito-temporal sensors (see methods), ERFs averaged across 100–200 ms were entered into an initial 2×2×2×2 ANOVA (hemisphere x drug group x probability x reward magnitude, see methods). This analysis revealed a main effect of probability (*F*(1,33) = 4.79, *p* = 0.036), a significant interaction probability x drug group (*F*(1,33) = 6.49, *p* = 0.016), hemisphere x probability (*F*(1,33) = 5.86, *p* = 0.021), and a three-way interaction of hemisphere x probability x drug group (*F*(1,33) = 8.04, *p* = 0.008). There were no significant main effects of hemisphere (*F*(1,33) = 2.00, *p* = 0.167), reward magnitude (*F*(1,33) = 0.36, *p* = 0.555) or drug group (*F*(1,33) = 0.22, *p* = 0.646) and no other significant interactions (all p's>0.05). Further exploration of the interaction hemisphere x probability x drug group revealed a main effect of probability (*F*(1,33) = 5.37, *p* = 0.027) and a probability x drug group interaction (*F*(1,33) = 7.33, *p* = 0.011) for the sensor cluster of the right but not the left (all p's>0.05) hemisphere. Furthermore, the interaction probability x drug group at the right sensor cluster was driven by significantly more negative deflections to high probability cues (i.e., 0.7) compared to low probability cues (i.e., 0.3) for the placebo group (*t*(16) = −3.94, *p* = 0.001). In contrast, there was no probability effect (i.e. ERF differences to low probability cues vs. high probability cues) for the levodopa group (*t*(17) = 0.26, *p* = 0.801) (Figure 2).

Visual inspection of ERFs during reward anticipation suggested the existence of reward probability effects at an even earlier point in time (i.e., 90–110 ms, see Figure 2B). Further analysis of neural activity in this time window, however, revealed no significant effects of reward anticipation (for details see Analysis S2).

Finally, additional analyses were carried out to ensure that monetary rewards received during a previous experiment (i.e., [28]) had no bearing on the neural effects of interest in the current study (see Analysis S1 for further details).

**Figure 3:**
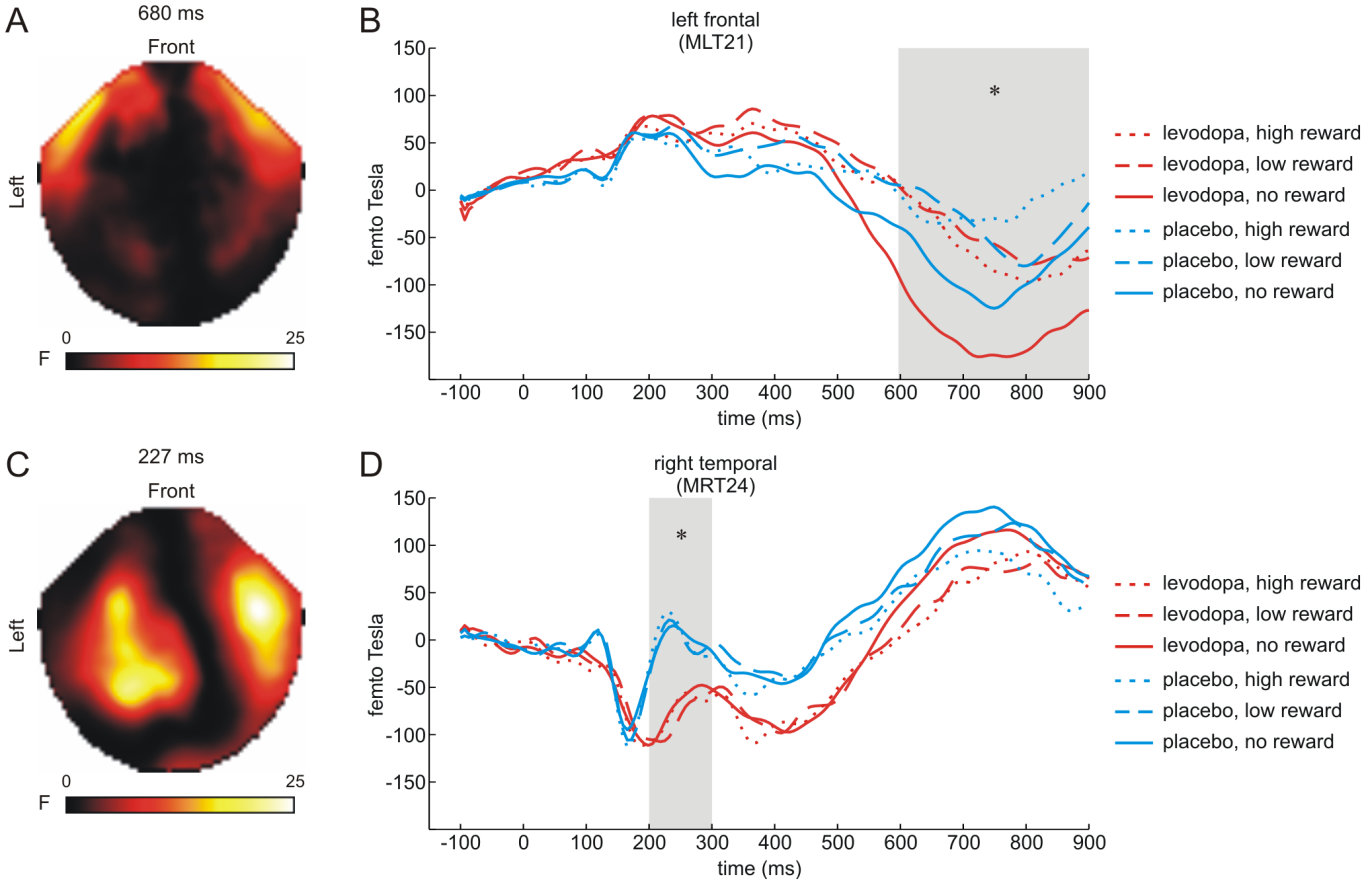
ERFs for reward outcome. SPM-based analyses across all sensors and time-points revealed a main effect of outcome magnitude (high/1.00€, low/0.30€, no/0.00€ reward outcome) at left temporal sensors (peak at 680 ms; nearest sensor: MLT21) (A). Furthermore, main effects of drug group (levodopa, placebo) arose at right temporal sensors (peak at 227 ms; nearest sensor: MRT24) (C). The main effect of outcome magnitude was driven by more negative deflections for the “no reward” condition compared to both the “low reward” and “high reward” condition in the time window of 600 to 900 ms after stimulus onset (B). The main effect of drug group was due to more negative deflections in the levodopa group compared to the placebo group between 200 and 300 ms (D). Asterisks indicate statistical significant main effects (*p*<0.05).

In a second step, we assessed the influence of levodopa on processing reward outcome by means of a 3×2 ANOVA across all sensors and time-points (i.e., −100–1000 ms, see methods section) as implemented in SPM8. Since we had no specific *a priori* hypotheses, all statistical parametric maps were family-wise error (FWE) corrected at a statistical threshold of *p*<0.05 in order to account for multiple statistical comparisons. This ANOVA revealed a main effect of magnitude at left fronto-temporal sensors peaking at 680 ms after stimulus onset (Figure 3A; cluster size k = 294 voxels); nearest sensor: MLT21; *p*<0.05 FWE-corrected). Closer inspection revealed that this main effect was due to more negative deflections to no reward outcome (i.e., €0.00) compared to both high (i.e., €1.00) (*t*(34) = 4.26, *p*<0.001) and low outcomes (i.e., €0.30) (*t*(34) = 3.98, *p*<0.001) (Figure 3B). There were no significant differences in the ERFs to high and low outcomes (*t*(34) = −0.47, *p* = 0.645). Moreover, there was a main effect of drug group (F-contrast) at right temporal sensors peaking at 227 ms (Figure 3C; cluster size k = 58 voxels; nearest sensor: MRT24; *p*<0.05 FWE-corrected). It was driven by more negative deflections for the levodopa group compared to the placebo group in the time window ranging from ∼200 to 300 ms after stimulus onset (*t*(33) = −2.07, *p* = 0.046) (Figure 3D). No interactions between drug group and outcome magnitude survived family-wise error correction (all *p*'s>0.05). Similarly, there were no statistically significant effects during baseline period (p>0.05, FWE-corrected).

### Time-frequency responses

Oscillatory responses (power) were assessed for cues (2×2×2 ANOVA; drug group x probability x reward magnitude, see methods) and outcomes (3×2 ANOVA; outcome magnitude x drug group, see methods). Here, we focused on low beta (13–20 Hz) and high beta (20–30 Hz) frequency ranges (see introduction).

**Figure 4:**
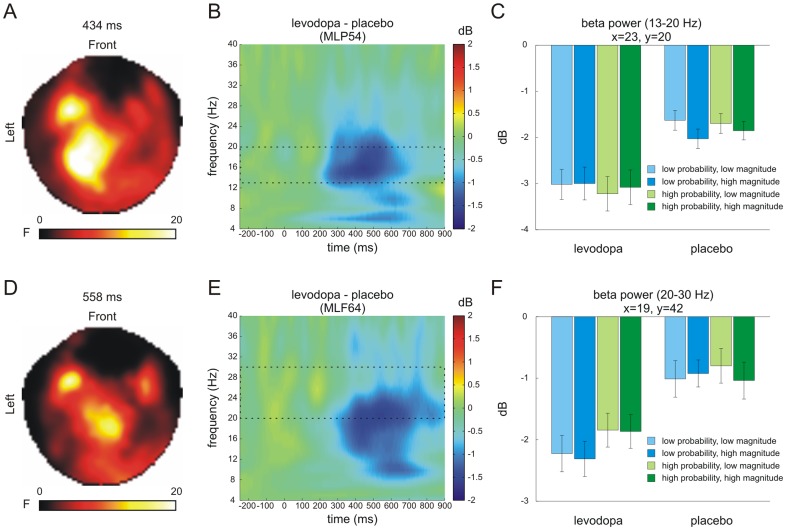
Time-frequency effects for reward predicting cues. SPM-based analyses across all sensors and time-points revealed main effects of drug group (levodopa, placebo) at left parietal sensors in the low beta band (peak at 434 ms; nearest sensor: MLP54) (A) and left frontal sensors for the high beta band (peak at 558 ms; nearest sensor: MLF64) following cue presentation (D). Both effects were driven by lower beta power in the levodopa group compared to the placebo group (C and F). Left columns show statistical parametric maps of the F-statistics, middle columns show the time-frequency plots as extracted from the nearest channel, and right columns show the effects for each condition at the peak time point as extracted from voxel space. Dashed boxes in B and E indicate the low (13–20 Hz) and high (20–30 Hz) beta band, respectively. Error bars in C and F denote one standard error of the mean (SEM).

For cues, we observed a main effect of drug group in the low beta band at left parietal sensors peaking at 434 ms after stimulus onset (Figure 4A; cluster size k = 7164 voxels; nearest sensor: MLP54; *p*<0.05 FWE-corrected). It was based on significantly reduced power for the levodopa group in contrast to the placebo group (*t*(33) = −3.30, *p* = 0.002). For high beta (20–30 Hz), the ANOVA revealed a main effect of drug group at frontal sensors with a peak at 558 ms after stimulus onset (Figure 4D; cluster size k = 68 voxels; nearest sensor: MLF64; *p*<0.05 FWE-corrected). Similar to the low beta band, oscillatory responses for the levodopa group were significantly lower in contrast to the placebo group (*t*(33) = −3.14, *p* = 0.004). Closer inspection of this effect revealed a main effect (F-contrast) of probability at the same voxel (*F*(1,33) = 5.88, *p* = 0.021) which was driven by significantly lower oscillatory power for low probability cues (i.e., 0.3) compared to high probability cues (i.e., 0.7) (*t*(34) = −2.39, *p* = 0.023) (Figure 4F). There was no statistically significant interaction in either frequency band for cue related oscillatory activity and no main effects or interactions were observed for the baseline period (all *p*'s>0.05 after FWE-correction).

**Figure 5:**
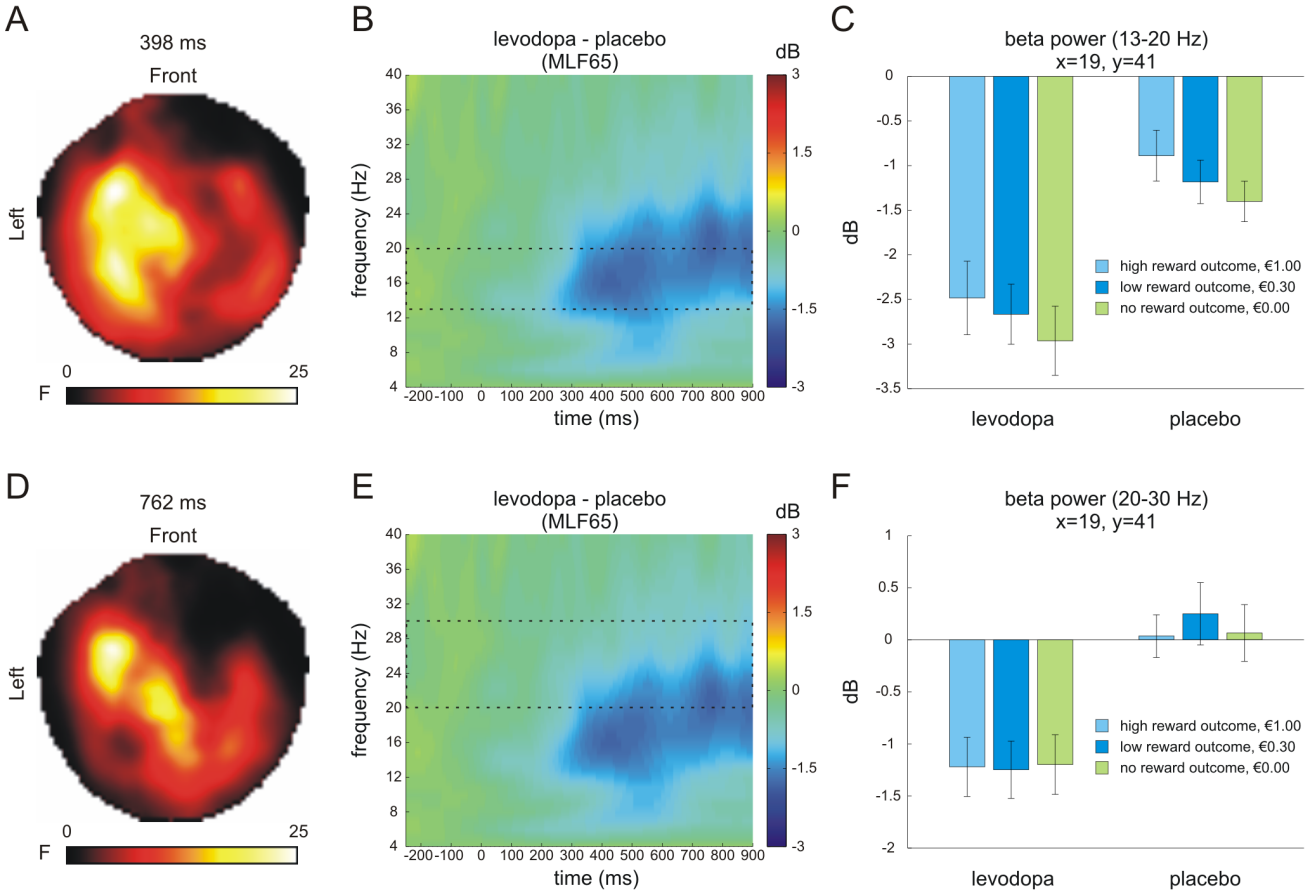
Time-frequency effects for reward outcome. SPM-based analyses across all sensors and time-points revealed main effects of drug group (levodopa, placebo) at left frontal sensors for both the low beta band (peak at 398 ms; nearest sensor: MLF65) (A) and high beta band (peak at 762 ms; same nearest sensor) following outcome presentation (D). Both effects were driven by reduced beta power in the levodopa group compared to the placebo group (C and F). In addition, power in the low beta band decreased as a function of outcome magnitude (i.e., from high to low magnitude) at the peak voxel showing the group effect (C). Left columns show statistical parametric maps of the F-statistics, middle columns show the time-frequency plots as extracted from the nearest channel, and right columns show the effects for each condition at the peak time point as extracted from voxel space. Dashed boxes in B and E indicate the low (13–20 Hz) and high (20–30 Hz) beta band, respectively. Error bars in C and F denote one SEM.

For reward outcome, there was a main effect of drug group in the low beta frequency band at left frontal sensors, peaking at 398 ms after stimulus onset (Figure 5A; cluster size k = 1084 voxels; nearest sensor: MLF65; *p*<0.05 FWE-corrected). This effect was driven by lower power for the levodopa compared to the placebo group (*t*(33) = −3.63, *p* = 0.001). Further inspection of this effect also revealed a main effect of outcome magnitude (F-contrast) at the same peak voxel (*F*(2,66) = 5.16, *p* = 0.008) (Figure 5C). Here, no reward outcomes (i.e., €0.00) were associated with lower power compared to high reward outcomes (i.e., €1.00) (*t*(34) = 3.53, *p* = 0.001) suggesting a linearly decrease with outcome magnitude (linear trend; *p* = 0.001). Finally, we observed a main effect of drug group in the high beta frequency range, again at frontal sensors but at a later point in time with a peak at 762 ms (Figure 5D; cluster size k = 113 voxels; nearest sensor: MLF65; *p*<0.05 FWE-corrected) that was driven by reduced oscillatory beta power for the levodopa group compared to the placebo group (*t*(33) = −3.96, *p*<0.001). No further main effects or interactions survived family-wise error correction, including baseline (all *p*'s>0.05).

## Discussion

We investigated the neural mechanisms of early reward anticipation as well as reward outcome processing and their link to dopaminergic modulation. As hypothesized, and in line with previous findings [18], [19], reward probability was signaled in the ERFs at occipital sensors peaking at ∼150 ms after stimulus onset. Remarkably, this early effect was absent in the levodopa group (Figure 2), indicating that dopamine (indirectly) modulates the distinction of reward probabilities in the visual cortex. Furthermore, administration of levodopa resulted in lower oscillatory beta power in response to cues and outcome independent of their predictive features (i.e., probability and reward magnitude) or outcome magnitude (i.e., high, low, or no reward outcome) (Figures 4 and 5).

### Reward anticipation – ERFs

A wide range of electrophysiological studies in animals and fMRI studies in humans have revealed a network of both cortical and subcortical brain regions that code forthcoming rewards [13], [16], [35], [36]. Most prominently, it includes the substantia nigra/ventral tegmental area (SN/VTA), basal ganglia as well as the prefrontal and parietal cortex. More recently, however, a growing body of literature has provided evidence for the notion that reward signals are represented beyond these mesolimbic and cortical areas. Particularly, in an fMRI study in humans, the signaling of anticipated reward probabilities has been found to extend to the superior occipital gyrus [37]. That is, activity in these visual brain regions scaled with increasing reward likelihood. Furthermore, another fMRI study reported that subjective value correlated with blood oxygen level dependent (BOLD) activity in the middle occipital gyrus in a probabilistic context [38].

Although our understanding of visual sensory areas in processing reward expectation is still limited, recent work gave some important insights. Specifically, MEG [18], [19], EEG [20] and combined M/EEG [21] studies have found modulations of ERFs and event-related potentials (ERPs), respectively, in response to reward predicting cues at temporo-occipital [18] and parieto-occipital sensors [20], [21]. While these studies demonstrated magnitude [20] and probability [18], [19] effects during reward anticipation, we only observed probability but not magnitude effects at occipital sensors (Figure 2). This dissociation might be due to design complexity (see below) and argues against a common neural mechanism for probability and magnitude processing in the visual cortex.

Another important finding of our study is that stimulating the dopaminergic system by levodopa significantly reduced the early probability effect at occipital sensors (Figure 2B and C), which points towards a close link. Although we do not have any direct evidence in our data, physiologically, this effect is likely to be mediated via the prefrontal cortex. Indeed, previous animal research demonstrated that dopamine not only regulates prefrontal sensory signals [39], but also projections that control visual signals in the occipital cortex [8]. Therefore, the prefrontal cortex seems to prepare visual brain regions for behaviorally relevant sensory events via dopaminergic neuromodulation. Why dopaminergic stimulation down-regulates the probability effect rather than enhances it, remains currently unclear. One possibility is that the relationship between prefrontal dopamine levels and neural processing (and cognition) follows an inverted u-shaped relationship [40], [41]. That means, moderate levels of prefrontal dopamine drive neural and cognitive functions but too low or excessive dopamine levels (as in our study) might impair them.

The absence of a magnitude effect in the ERFs at occipital sensors is at odds with previous work. For instance, fMRI studies in humans have found that activity in the visual cortex can be modulated by expected reward magnitude [42], [43]. This was paralleled by electrophysiological recordings demonstrating ERP/ERF differences for cues signaling high vs. low rewards [20] and reward vs. no reward [21], respectively. One possible explanation for our diverging results could either relate to the rather small rewards (€0.30, €1.00) or, more likely, the complexity of the task. That means, in our study, both magnitude and probability was varied while previous M/EEG studies, that focused on reward anticipation, only manipulated one of these two factors [19]–[21]. A similar explanation might hold true for the absence of a magnitude effect in the beta band (see below).

### Reward anticipation – beta power

As expected, beta power at frontal sensors signaled reward probability (Figure 4D), which further implies a role of high (20–30 Hz) beta band oscillations in reward processing. From a physiological point of view, beta band oscillations may reflect the binding of the distributed brain regions that are involved in reward anticipation [44], [45]. This possibility receives support from a recent EEG study [46] providing evidence for a link between beta power and dopaminergic neuromodulation by investigating genetic variability of catechol-*O*-methyltranferase (COMT). More precisely, in their study, the authors showed that beta power was increased for gains in participants homozygous for the COMT ValVal allele as compared to participants homozygous for the MetMet allele, which is associated with low enzyme activity and, in turn, increased levels of tonic dopamine [47].

In support of a link between dopamine and beta band oscillations during reward anticipation, our data show that elevated levels of dopamine reduce beta power at left parietal and frontal sensors (Figure 4). However, there was no differential effect of drug on probability or magnitude, which is partly in line with the absence of a drug effect on behavior. That means, despite the overall high task performance (Tables 1 and 2), dopaminergic stimulation did not affect reaction times or accuracy. This could be due to ceiling effects at the behavioral level or, alternatively, to inappropriate dosages of levodopa.

### Reward outcome – ERFs

Previous studies in humans have reported characteristic ERP and ERF components in the time-range of 200–300 ms, such as the error-related negativity (ERN) [48], [49], P300 [50], feedback-related negativity (FRN) [51] and the mFRN (the MEG counterpart of the EEG FRN) [52], to be sensitive to outcome signals. These components have been linked to various cognitive processes related to the evaluation of behavior, including monitoring erroneous performance in case of the ERN [48] or feedback responses in case of the FRN [51] and P300 [53]. In comparison to these earlier findings, in our study, we detected a significant effect of reward magnitude in the ERFs for outcome at frontal sensors only after ∼600 ms (Figure 3A and B) which, contrary to our expectations, was not modulated by levodopa administration. As mentioned above, our design differs from most previous studies since it varies both magnitude and probability. Therefore, we speculate that task complexity could modulate the temporal dynamics of outcome processing.

Even though our data do not provide evidence for a dopaminergic modulation of the rather late ERF outcome signals, we do not take these data to argue against an involvement of dopamine in the processing of reward feedback. Instead, it seems more likely that MEG is not suitable to detect signals from those deep subcortical brain regions [54], such as the ventral striatum or SN/VTA, that may have coded outcome magnitude [3], [55].

We observed a significant main effect of drug group on outcome processing at right temporal sensors with a peak at ∼200–300 ms after stimulus onset (Figure 3C and D). This effect was driven by stronger negative ERFs in the levodopa group irrespective of magnitude. Based on previous reports of aberrant signal processing under elevated levels of dopamine [56], [57], we suggest that the observed effect most likely reflects enhanced stimulus salience [58]. In support of this idea, we could recently show that levodopa enhances ERFs to several stimulus classes, including novel and repeated items [27] as well as rewarded and unrewarded images [28]. Similarly, others [59] have demonstrated a close link between striatal dopamine release and prefrontal salience processing. However, in our study there was no direct relationship between the main effect of drug and behavior, which leaves the interpretation of enhanced salience speculative.

### Reward outcome – beta power

With regard to oscillatory responses, we observed increases in low beta power (13–20 Hz) as a function of magnitude at left frontal sensors starting at around 300 ms after stimulus onset (Figure 5A–C). This observation is consistent with previous findings [21]–[23], [60] and underlines the role of beta oscillations not only in reward anticipation but also outcome processing. However, there was no differential effect of levodopa on outcome related beta activation but a main effect of drug group at frontal sensors (Figure 5). That means, levodopa administration led to a general decrease of beta power both in the low (13–20 Hz) and high (20–30 Hz) beta band irrespective of the delivered reward magnitude.

Although previous work suggests that reward can increase response vigor possibly via enhanced dopamine release [61], [62], we did not observe any behavioral effects of reward magnitude or levodopa on reaction times or hit rates at outcome. Similar to the anticipation phase, we suggest that this might relate to task complexity, ceiling effects or the administered dosage. Since these factors cannot be disentangled on basis of our present data, the exact nature of a possible link remains unresolved for the time being.

Finally, we acknowledge that between-subjects designs may be less sensitive as compared to within-subjects designs. Therefore, the weak (or even absent) effects of levodopa on reward anticipation, outcome processing and cognition may be due lower statistical power, which should be considered in future studies.

## Summary

We can show that the human brain signals the anticipation of monetary reward as early as ∼100 ms after stimulus onset. Importantly, these effects emerged at occipital sensors and were modulated by levodopa. More precisely, while reward probability was rapidly signaled at moderate levels of available dopamine (i.e., in the placebo group), these responses were abolished at elevated dopamine levels (i.e., after levodopa administration). Similarly, reward probability was signaled in the high beta band but there was only a general effect of levodopa on beta power irrespective of reward probability and magnitude. Taken together, our data provide further evidence for a close link between dopaminergic neuromodulation, oscillatory activity in the beta band and early visual reward signals.

## Supporting Information

Table S1 **Subjective ratings.**
(DOCX)

Table S2 **Nicotine consumption of participants.**
(DOCX)

Analysis S1 **Assessment of potential drug side-effects and group differences.**
(DOCX)

Analysis S2 **Supplemental analyses of ERFs during reward anticipation.**
(DOCX)

## References

[1] Schultz W (2007) Multiple dopamine functions at different time courses. Annu Rev Neurosci 30: 259–288 10.1146/annurev.neuro.28.061604.135722 17600522

[2] Fiorillo CD, Tobler PN, Schultz W (2003) Discrete coding of reward probability and uncertainty by dopamine neurons. Science 299: 1898–1902 10.1126/science.1077349 12649484

[3] Tobler PN, Fiorillo CD, Schultz W (2005) Adaptive coding of reward value by dopamine neurons. Science 307: 1642–1645 10.1126/science.1105370 15761155

[4] Watanabe M (1996) Reward expectancy in primate prefrontal neurons. Nature 382: 629–632 10.1038/382629a0 8757133

[5] Kawagoe R, Takikawa Y, Hikosaka O (1998) Expectation of reward modulates cognitive signals in the basal ganglia. Nat Neurosci 1: 411–416 10.1038/1625 10196532

[6] Platt ML, Glimcher PW (1999) Neural correlates of decision variables in parietal cortex. Nature 400: 233–238 10.1038/22268 10421364

[7] Shuler MG, Bear MF (2006) Reward timing in the primary visual cortex. Science 311: 1606–1609 10.1126/science.1123513 16543459

[8] Noudoost B, Moore T (2011) Control of visual cortical signals by prefrontal dopamine. Nature 474: 372–375 10.1038/nature09995 21572439 PMC3117113

[9] Cohen MX (2008) Neurocomputational mechanisms of reinforcement-guided learning in humans: a review. Cogn Affect Behav Neurosci 8: 113–125. 18589502 10.3758/cabn.8.2.113

[10] Dillon DG, Holmes AJ, Jahn AL, Bogdan R, Wald LL, et al (2008) Dissociation of neural regions associated with anticipatory versus consummatory phases of incentive processing. Psychophysiology 45: 36–49 10.1111/j.14698986.2007.00594.x 17850241 PMC2156200

[11] Ernst M, Nelson EE, McClure EB, Monk CS, Munson S, et al (2004) Choice selection and reward anticipation: an fMRI study. Neuropsychologia 42: 1585–1597 10.1016/j.neuropsychologia.2004.05.011 15327927

[12] Galvan A, Hare TA, Davidson M, Spicer J, Glover G, et al (2005) The role of ventral frontostriatal circuitry in reward-based learning in humans. J Neurosci 25: 8650–8656 10.1523/JNEUROSCI.2431-05.2005 16177032 PMC6725514

[13] Knutson B, Cooper JC (2005) Functional magnetic resonance imaging of reward prediction. Curr Opin Neurol 18: 411–417. 16003117 10.1097/01.wco.0000173463.24758.f6

[14] Knutson B, Adams CM, Fong GW, Hommer D (2001) Anticipation of increasing monetary reward selectively recruits nucleus accumbens. J Neurosci 21: RC159. 11459880 10.1523/JNEUROSCI.21-16-j0002.2001 PMC6763187

[15] Knutson B, Westdorp A, Kaiser E, Hommer D (2000) FMRI visualization of brain activity during a monetary incentive delay task. Neuroimage 12: 20–27 10.1006/nimg.2000.0593 10875899

[16] O′Doherty JP (2004) Reward representations and reward-related learning in the human brain: insights from neuroimaging. Curr Opin Neurobiol 14: 769–776 10.1016/j.conb.2004.10.016 15582382

[17] Yacubian J, Gläscher J, Schroeder K, Sommer T, Braus DF, et al (2006) Dissociable systems for gain- and loss-related value predictions and errors of prediction in the human brain. J Neurosci 26: 9530–9537 10.1523/JNEUROSCI.2915-06.2006 16971537 PMC6674602

[18] Bunzeck N, Guitart-Masip M, Dolan RJ, Düzel E (2011) Contextual novelty modulates the neural dynamics of reward anticipation. J Neurosci 31: 12816–12822 10.1523/JNEUROSCI.0461-11.2011 21900560 PMC3192314

[19] Thomas J, Vanni-Mercier G, Dreher J-C (2013) Neural dynamics of reward probability coding: a Magnetoencephalographic study in humans. Front Neurosci 7: 214 10.3389/fnins.2013.00214 24302894 PMC3831091

[20] Gruber MJ, Otten LJ (2010) Voluntary Control over Prestimulus Activity Related to Encoding. The Journal of Neuroscience 30: 9793–9800 10.1523/JNEUROSCI.0915-10.2010 20660262 PMC2929460

[21] Doñamayor N, Schoenfeld MA, Münte TF (2012) Magneto- and electroencephalographic manifestations of reward anticipation and delivery. Neuroimage 62: 17–29 10.1016/j.neuroimage.2012.04.038 22561022

[22] HajiHosseini A, Rodríguez-Fornells A, Marco-Pallarés J (2012) The role of beta-gamma oscillations in unexpected rewards processing. Neuroimage 60: 1678–1685 10.1016/j.neuroimage.2012.01.125 22330314

[23] Marco-Pallares J, Cucurell D, Cunillera T, García R, Andrés-Pueyo A, et al (2008) Human oscillatory activity associated to reward processing in a gambling task. Neuropsychologia 46: 241–248 10.1016/j.neuropsychologia.2007.07.016 17804025

[24] Kawasaki M, Yamaguchi Y (2013) Frontal theta and beta synchronizations for monetary reward increase visual working memory capacity. Soc Cogn Affect Neurosci 8: 523–530 10.1093/scan/nss027 22349800 PMC3682435

[25] Bunzeck N, Guitart-Masip M, Dolan RJ, Duzel E (2013) Pharmacological Dissociation of Novelty Responses in the Human Brain. Cereb Cortex 10.1093/cercor/bhs420 PMC3977623 23307638

[26] Guitart-Masip M, Chowdhury R, Sharot T, Dayan P, Duzel E, et al (2012) Action controls dopaminergic enhancement of reward representations. Proc Natl Acad Sci USA 109: 7511–7516 10.1073/pnas.1202229109 22529363 PMC3358848

[27] Eckart C, Bunzeck N (2013) Dopamine modulates processing speed in the human mesolimbic system. NeuroImage 66: 293–300 10.1016/j.neuroimage.2012.11.001 23142277

[28] Apitz T, Bunzeck N (2013) Dopamine controls the neural dynamics of memory signals and retrieval accuracy. Neuropsychopharmacology. doi:10.1038/npp.2013.141 10.1038/npp.2013.141 PMC3799060 23728140

[29] Nyholm D, Lewander T, Gomes-Trolin C, Bäckström T, Panagiotidis G, et al (2012) Pharmacokinetics of levodopa/carbidopa microtablets versus levodopa/benserazide and levodopa/carbidopa in healthy volunteers. Clin Neuropharmacol 35: 111–117 10.1097/WNF.0b013e31825645d1 22549097

[30] Worsley KJ, Taylor JE, Tomaiuolo F, Lerch J (2004) Unified univariate and multivariate random field theory. Neuroimage 23 Suppl 1S189–195 10.1016/j.neuroimage.2004.07.026 15501088

[31] Kilner JM, Friston KJ (2010) Topological inference for EEG and MEG. The Annals of Applied Statistics 4: 1272–1290 10.1214/10-AOAS337

[32] Hsieh L-T, Ekstrom AD, Ranganath C (2011) Neural oscillations associated with item and temporal order maintenance in working memory. J Neurosci 31: 10803–10810 10.1523/JNEUROSCI.0828-11.2011 21795532 PMC3164584

[33] Khader PH, Jost K, Ranganath C, Rösler F (2010) Theta and alpha oscillations during working-memory maintenance predict successful long-term memory encoding. Neurosci Lett 468: 339–343 10.1016/j.neulet.2009.11.028 19922772 PMC3951969

[34] Litvak V, Mattout J, Kiebel S, Phillips C, Henson R, et al (2011) EEG and MEG data analysis in SPM8. Comput Intell Neurosci 2011: 852961 10.1155/2011/852961 21437221 PMC3061292

[35] Bunzeck N, Doeller CF, Dolan RJ, Duzel E (2012) Contextual interaction between novelty and reward processing within the mesolimbic system. Hum Brain Mapp 33: 1309–1324 10.1002/hbm.21288 21520353 PMC3498733

[36] Schultz W (2004) Neural coding of basic reward terms of animal learning theory, game theory, microeconomics and behavioural ecology. Curr Opin Neurobiol 14: 139–147 10.1016/j.conb.2004.03.017 15082317

[37] Guitart-Masip M, Bunzeck N, Stephan KE, Dolan RJ, Düzel E (2010) Contextual novelty changes reward representations in the striatum. J Neurosci 30: 1721–1726 10.1523/JNEUROSCI.5331-09.2010 20130181 PMC2838369

[38] Peters J, Büchel C (2009) Overlapping and distinct neural systems code for subjective value during intertemporal and risky decision making. J Neurosci 29: 15727–15734 10.1523/JNEUROSCI.3489-09.2009 20016088 PMC6666169

[39] Jacob SN, Ott T, Nieder A (2013) Dopamine regulates two classes of primate prefrontal neurons that represent sensory signals. J Neurosci 33: 13724–13734 10.1523/JNEUROSCI.0210-13.2013 23966694 PMC6618653

[40] Cools R, D′Esposito M (2011) Inverted-U-shaped dopamine actions on human working memory and cognitive control. Biol Psychiatry 69: e113–125 10.1016/j.biopsych.2011.03.028 21531388 PMC3111448

[41] Goldman-Rakic P, Muly I, Williams G (2000) D1 receptors in prefrontal cells and circuits. Brain Research Reviews 31: 295–301 10.1016/S0165-0173(99)00045-4 10719156

[42] Engelmann JB, Damaraju E, Padmala S, Pessoa L (2009) Combined effects of attention and motivation on visual task performance: transient and sustained motivational effects. Front Hum Neurosci 3: 4 10.3389/neuro.09.004.2009 19434242 PMC2679199

[43] Weil RS, Furl N, Ruff CC, Symmonds M, Flandin G, et al (2010) Rewarding feedback after correct visual discriminations has both general and specific influences on visual cortex. J Neurophysiol 104: 1746–1757 10.1152/jn.00870.2009 20660419 PMC2944687

[44] Buzsáki G, Draguhn A (2004) Neuronal oscillations in cortical networks. Science 304: 1926–1929 10.1126/science.1099745 15218136

[45] Varela F, Lachaux JP, Rodriguez E, Martinerie J (2001) The brainweb: phase synchronization and large-scale integration. Nat Rev Neurosci 2: 229–239 10.1038/35067550 11283746

[46] Marco-Pallarés J, Cucurell D, Cunillera T, Krämer UM, Càmara E, et al (2009) Genetic variability in the dopamine system (dopamine receptor D4, catechol-O-methyltransferase) modulates neurophysiological responses to gains and losses. Biol Psychiatry 66: 154–161 10.1016/j.biopsych.2009.01.006 19251248

[47] Bilder RM, Volavka J, Lachman HM, Grace AA (2004) The catechol-O-methyltransferase polymorphism: relations to the tonic-phasic dopamine hypothesis and neuropsychiatric phenotypes. Neuropsychopharmacology 29: 1943–1961 10.1038/sj.npp.1300542 15305167

[48] Holroyd CB, Coles MGH (2002) The neural basis of human error processing: reinforcement learning, dopamine, and the error-related negativity. Psychol Rev 109: 679–709. 12374324 10.1037/0033-295X.109.4.679

[49] Nieuwenhuis S, Yeung N, Holroyd CB, Schurger A, Cohen JD (2004) Sensitivity of electrophysiological activity from medial frontal cortex to utilitarian and performance feedback. Cereb Cortex 14: 741–747 10.1093/cercor/bhh034 15054053

[50] Wu Y, Zhou X (2009) The P300 and reward valence, magnitude, and expectancy in outcome evaluation. Brain Research 1286: 114–122 10.1016/j.brainres.2009.06.032 19539614

[51] Yu R, Zhou X (2006) Brain potentials associated with outcome expectation and outcome evaluation. Neuroreport 17: 1649–1653 10.1097/01.wnr.0000236866.39328.1d 17001286

[52] Doñamayor N, Marco-Pallarés J, Heldmann M, Schoenfeld MA, Münte TF (2011) Temporal dynamics of reward processing revealed by magnetoencephalography. Hum Brain Mapp 32: 2228–2240 10.1002/hbm.21184 21305665 PMC6870214

[53] Wu K, O′Keeffe D, Politis M, O′Keeffe GC, Robbins TW, et al (2012) The catechol-O-methyltransferase Val(158)Met polymorphism modulates fronto-cortical dopamine turnover in early Parkinson's disease: a PET study. Brain 135: 2449–2457 10.1093/brain/aws157 22843413

[54] Hillebrand A, Barnes GR (2002) A quantitative assessment of the sensitivity of whole-head MEG to activity in the adult human cortex. Neuroimage 16: 638–650. 12169249 10.1006/nimg.2002.1102

[55] Haber SN, Knutson B (2010) The reward circuit: linking primate anatomy and human imaging. Neuropsychopharmacology 35: 4–26 10.1038/npp.2009.129 19812543 PMC3055449

[56] Kapur S (2003) Psychosis as a state of aberrant salience: a framework linking biology, phenomenology, and pharmacology in schizophrenia. Am J Psychiatry 160: 13–23. 12505794 10.1176/appi.ajp.160.1.13

[57] Lodge DJ, Grace AA (2007) Aberrant hippocampal activity underlies the dopamine dysregulation in an animal model of schizophrenia. J Neurosci 27: 11424–11430 10.1523/JNEUROSCI.2847-07.2007 17942737 PMC6673030

[58] Berridge KC (2007) The debate over dopamine's role in reward: the case for incentive salience. Psychopharmacology (Berl) 191: 391–431 10.1007/s00213-006-0578-x 17072591

[59] Bhattacharyya S, Crippa JA, Allen P, Martin-Santos R, Borgwardt S, et al (2012) Induction of psychosis by Δ9-tetrahydrocannabinol reflects modulation of prefrontal and striatal function during attentional salience processing. Arch Gen Psychiatry 69: 27–36 10.1001/archgenpsychiatry.2011.161 22213786

[60] Cohen MX, Elger CE, Ranganath C (2007) Reward expectation modulates feedback-related negativity and EEG spectra. Neuroimage 35: 968–978 10.1016/j.neuroimage.2006.11.056 17257860 PMC1868547

[61] Beierholm U, Guitart-Masip M, Economides M, Chowdhury R, Düzel E, et al (2013) Dopamine modulates reward-related vigor. Neuropsychopharmacology 38: 1495–1503 10.1038/npp.2013.48 23419875 PMC3682144

[62] Niv Y, Daw ND, Joel D, Dayan P (2007) Tonic dopamine: opportunity costs and the control of response vigor. Psychopharmacology (Berl) 191: 507–520 10.1007/s00213-006-0502-4 17031711

