# Supplementary material for: Early Effects of Reward Anticipation Are Modulated by Dopaminergic Stimulation
Source: PLoS One. 2014 Oct 6;9(10):e108886. doi: 10.1371/journal.pone.0108886 (PMC4186816; doi:10.1371/journal.pone.0108886)
Supplement: Table S1 — Subjective ratings. (DOCX) [file pone.0108886.s001.docx]

**Table S1**. **Subjective ratings** (ranging from 0 – 7). T1 indicates time-point before drug administration, T2 = 45 min after drug intake, T3 = after the MEG scan (~ 2 h after drug intake). PL = Placebo, LV = Levodopa. M: mean (SD: standard deviation).

|  | **T1** | | **T2** | | **T3** | |
| --- | --- | --- | --- | --- | --- | --- |
|  | PL | LV | PL | LV | PL | LV |
|  | M (SD) | M (SD) | M (SD) | M (SD) | M (SD) | M (SD) |
| **Subjective rating scale** |  |  |  |  |  |  |
| **Alert/** | 1.28 | 1.09 | 0.97 | 1.44 | 1.91 | 2.42 |
| **Drowsy** | (1.28) | (1.21) | (0.89) | (1.50) | (2.09) | (1.74) |
| **Calm/** | 1.62 | 1.67 | 1.65 | 1.70 | .79 | 1.44 |
| **Excited** | (1.13) | (1.45) | (1.41) | (1.67) | (.98) | (1.38) |
| **Strong/** | 1.26 | 1.14 | 1.19 | 1.13 | 1.29 | 1.67 |
| **Feeble** | (1.01) | (1.22) | (0.95) | (1.29) | (1.27) | (1.55) |
| **Clear-Headed/** | .74 | .55 | .81 | .80 | 1.51 | 1.18 |
| **Muzzy** | (.92) | (.52) | (1.08) | (.67) | (1.64) | (1.01) |
| **Well-Coordinated/** | 1.46 | 1.39 | 1.2 | 1.52 | 1.66 | 1.95 |
| **Clumsy** | (1.19) | (1.14) | (1.17) | (1.26) | (1.49) | (1.53) |
| **Energetic/** | 1.62 | 1.67 | 1.30 | 1.58 | 1.82 | 2.09 |
| **Lethargic** | (.97) | (1.01) | (1.10) | (1.24) | (1.66) | (1.36) |
| **Contented/** | 1.16 | .83 | .86 | .87 | .95 | 1.11 |
| **Discontented** | (1.26) | (.94) | (1.19) | (.92) | (1.15) | (1.12) |
| **Tranquil/** | 1.13 | 1.13 | 1.11 | .98 | .94 | 1.44 |
| **Troubled** | (1.12) | (1.10) | (1.08) | (1.12) | (1.01) | (1.68) |
| **Quick-witted/** | .51 | .89 | .97 | .86 | .76 | 1.24 |
| **Mentally slow** | (.62) | (.78) | (1.04) | (.61) | (.82) | (1.06) |
| **Relaxed/** | 2.08 | 1.81 | 1.72 | 1.57 | 1.02 | 1.06 |
| **Tense** | (1.83) | (1.68) | (1.36) | (1.43) | (.98) | (1.05) |
| **Attentive/** | .87 | .95 | .74 | 1.05 | 1.21 | 1.45 |
| **Dreamy** | (.83) | (.85) | (.89) | (1.17) | (1.36) | (1.43) |
| **Proficient/** | .82 | .99 | 1.06 | .92 | 1.35 | 1.02 |
| **Incompetent** | (.96) | (.63) | (1.09) | (.75) | (1.27) | (.87) |
| **Happy/** | 1.04 | 1.08 | .81 | 1.24 | .95 | 1.04 |
| **Sad** | (1.14) | (1.08) | (1.02) | (1.29) | (1.18) | (1.25) |
| **Amicable/** | .60 | .81 | .75 | .71 | .69 | .75 |
| **Antagonistic** | (.82) | (1.00) | (1.05) | (.74) | (.93) | (.71) |
| **Interested/** | .79 | .57 | .83 | .68 | 1.36 | .94 |
| **Bored** | (1.02) | (.78) | (1.24) | (.85) | (1.44) | (.90) |
| **Gregarious/** | 2.56 | 2.44 | 2.66 | 2.18 | 2.46 | 2.33 |
| **Withdrawn** | (1.55) | (1.18) | (1.74) | (1.33) | (1.63) | (1.35) |
| **Secure/** | 1.11 | 1.04 | 1.11 | .96 | 1.01 | 1.02 |
| **Insecure** | (1.20) | (.98) | (1.09) | (1.10) | (1.01) | (1.23) |
