# Supplementary material for: Early Effects of Reward Anticipation Are Modulated by Dopaminergic Stimulation
Source: PLoS One. 2014 Oct 6;9(10):e108886. doi: 10.1371/journal.pone.0108886 (PMC4186816; doi:10.1371/journal.pone.0108886)
Supplement: Table S2 — Nicotine consumption of participants. (DOCX) [file pone.0108886.s002.docx]

| **Table S2. Nicotine consumption of participants**. Number of cigarettes consumed per day and total years of nicotine consumption. M: mean, SD: standard deviation. There were no differences between groups. | | | | |
| --- | --- | --- | --- | --- |
|  | Levodopa | | Placebo | |
|  | M | SD | M | SD |
| Cigarettes/day | *1.25* | *2.90* | *1.06* | *3.33* |
| Years of consumption | *3.06* | *4.92* | *2.10* | *6.00* |
