# Supplementary material for: Early Effects of Reward Anticipation Are Modulated by Dopaminergic Stimulation
Source: PLoS One. 2014 Oct 6;9(10):e108886. doi: 10.1371/journal.pone.0108886 (PMC4186816; doi:10.1371/journal.pone.0108886)
Supplement: Analysis S1 — Assessment of potential drug side-effects and group differences. (DOCX) [file pone.0108886.s003.docx]

**Analysis S1. Assessment of potential drug side-effects and group differences.** To assess possible side-effects of levodopa, scores of subjective well-being were entered into a 2x3 analyses of variance (ANOVA) with the between-subject factor group (levodopa, placebo) and the within-subject factor time (T1, T2, T3, see methods). There were no main effects of group and no drug x time interactions (all p>0.05).

A subset of participants had participated in an experiment that took place prior to the task of the present study (see methods section). To ensure that the amount of reward received in that previous experiment did not impact on the neural processing of reward information during the task of the present study, correlation analyses (Pearson’s *r*) between d-prime scores of the previous experiment (i.e., the discrimination performance, which was used to determine the amount of reward the participants received) and mean ERF amplitude differences during the reward anticipation phase (see Figure 2). This analysis revealed no statistical significant correlation between previously received reward and amplitude differences for either the levodopa (*r*=-0.305, *p*=0.250) or placebo group (*r*=0.102, *p*=0.697), and statistical comparison of the two correlation coefficients using Fisher z-transformation indicated no significant differences of these correlation indices (*z*=-1.084, *p*=0.279; two-tailed). A further correlation analysis, which comprised data for both groups also failed to reach statistical significance (*r*=0.019, *p*=0.918), indicating no relationship between monetary rewards received in the previous experiment and the neural processing of reward information during the anticipation phase.
