# Supplementary material for: Early Effects of Reward Anticipation Are Modulated by Dopaminergic Stimulation
Source: PLoS One. 2014 Oct 6;9(10):e108886. doi: 10.1371/journal.pone.0108886 (PMC4186816; doi:10.1371/journal.pone.0108886)
Supplement: Analysis S2 — Supplemental analyses of ERFs during reward anticipation. (DOCX) [file pone.0108886.s004.docx]

**Analysis S2. Supplemental analyses of ERFs during reward anticipation.** Visual inspection of ERFs during reward anticipation suggested the existence of reward probability effects at an even earlier point in time (i.e., 90–110 ms, see Figure 2). To further investigate these effects, we performed an additional ANOVA with the within-subject factors probability (0.3, 0.7), reward magnitude (€0.30, €1.00), time window (90–110 ms, 110–200 ms) and the between-subject factor drug group (levodopa, placebo). This analysis revealed a significant three-way interaction of time window x probability x drug group (*F*(1,33)=5.41, *p*=0.026). It was driven by a significant main effect of probability (*F*(1,33)=5.32, *p*=0.028) and a significant interaction of probability x drug group (*F*(1,33)=6.21, *p*=0.018) for the time window of 110–200 ms but not for the time window of 90–110 ms (main effect of probability: *F*(1,33)=0.16, *p*=0.695; interaction of probability x drug group: *F*(1,33)=3.56, *p*=0.068). The interaction of probability x drug group in the time window of 110–200 ms was based on more negative deflections to high probability cues (i.e., 0.7) compared to low probability cues (i.e., 0.3) for the placebo group (*t*(16)=-3.68, *p*=0.002). In contrast, there was no probability effect for the levodopa group (*t*(17)=0.12, *p*=0.904). We take the absence of a significant main effect of probability and interaction of the factors probability x drug group to argue against the presence of a robust modulation of neural activity during reward anticipation by dopamine prior to the original time window of interest.
